# T_1_-Mapping and extracellular volume estimates in pediatric subjects with Duchenne muscular dystrophy and healthy controls at 3T

**DOI:** 10.1186/s12968-020-00687-z

**Published:** 2020-12-10

**Authors:** Nyasha G. Maforo, Patrick Magrath, Kévin Moulin, Jiaxin Shao, Grace Hyun Kim, Ashley Prosper, Pierangelo Renella, Holden H. Wu, Nancy Halnon, Daniel B. Ennis

**Affiliations:** 1 Department of Radiological Sciences, University of California, Los Angeles, CA USA; 2 Physics and Biology in Medicine Interdepartmental Program, University of California, Los Angeles, CA USA; 3 Department of Bioengineering, University of California, Los Angeles, CA USA; 4 Department of Medicine, Division of Pediatric Cardiology, CHOC Children’s Hospital, Orange, CA USA; 5 Department of Pediatrics (Cardiology), University of California, Los Angeles, CA USA; 6 Department of Radiology, Stanford University, 1201 Welch Road, Room P264, Stanford, CA 94305-5488 USA; 7 Department of Biostatistics, University of California, Los Angeles, CA USA

**Keywords:** Duchenne muscular dystrophy, Cardiomyopathy, Cardiovascular magnetic resonance, Extracellular volume fraction, Late gadolinium enhancement, Myocardial remodeling, T_1_ mapping

## Abstract

**Background:**

Cardiovascular disease is the leading cause of death in patients with Duchenne muscular dystrophy (DMD)—a fatal X-linked genetic disorder. Late gadolinium enhancement (LGE) imaging is the current gold standard for detecting myocardial tissue remodeling, but it is often a late finding. Current research aims to investigate cardiovascular magnetic resonance (CMR) biomarkers, including native (pre-contrast) T_1_ and extracellular volume (ECV) to evaluate the early on-set of microstructural remodeling and to grade disease severity. To date, native T_1_ measurements in DMD have been reported predominantly at 1.5T. This study uses 3T CMR: (1) to characterize global and regional myocardial pre-contrast T_1_ differences between healthy controls and LGE + and LGE− boys with DMD; and (2) to report global and regional myocardial post-contrast T_1_ values and myocardial ECV estimates in boys with DMD, and (3) to identify left ventricular (LV) T_1_-mapping biomarkers capable of distinguishing between healthy controls and boys with DMD and detecting LGE status in DMD.

**Methods:**

Boys with DMD (N = 28, 13.2 ± 3.1 years) and healthy age-matched boys (N = 20, 13.4 ± 3.1 years) were prospectively enrolled and underwent a 3T CMR exam including standard functional imaging and T_1_ mapping using a modified Look-Locker inversion recovery (MOLLI) sequence. Pre-contrast T_1_ mapping was performed on all boys, but contrast was administered only to boys with DMD for post-contrast T_1_ and ECV mapping. Global and segmental myocardial regions of interest were contoured on mid LV T_1_ and ECV maps. ROI measurements were compared for pre-contrast myocardial T_1_ between boys with DMD and healthy controls, and for post-contrast myocardial T_1_ and ECV between LGE + and LGE− boys with DMD using a Wilcoxon rank-sum test. Results are reported as median and interquartile range (IQR). p-Values < 0.05 were considered significant. Receiver Operating Characteristic analysis was used to evaluate a binomial logistic classifier incorporating T_1_ mapping and LV function parameters in the tasks of distinguishing between healthy controls and boys with DMD, and detecting LGE status in DMD. The area under the curve is reported.

**Results:**

Boys with DMD had significantly increased global native T_1_ [1332 (60) ms vs. 1289 (56) ms; *p* = 0.004] and increased within-slice standard deviation (SD) [100 (57) ms vs. 74 (27) ms; *p* = 0.001] compared to healthy controls. LGE− boys with DMD also demonstrated significantly increased lateral wall native T_1_ [1322 (68) ms vs. 1277 (58) ms; *p* = 0.001] compared to healthy controls. LGE + boys with DMD had decreased global myocardial post-contrast T_1_ [565 (113) ms vs 635 (126) ms; *p* = 0.04] and increased global myocardial ECV [32 (8) % vs. 28 (4) %; *p* = 0.02] compared to LGE− boys. In all classification tasks, T_1_-mapping biomarkers outperformed a conventional biomarker, LV ejection fraction. ECV was the best performing biomarker in the task of predicting LGE status (AUC = 0.95).

**Conclusions:**

Boys with DMD exhibit elevated native T_1_ compared to healthy, sex- and age-matched controls, even in the absence of LGE. Post-contrast T_1_ and ECV estimates from 3T CMR are also reported here for pediatric patients with DMD for the first time and can distinguish between LGE + from LGE− boys. In all classification tasks, T_1_-mapping biomarkers outperform a conventional biomarker, LVEF*.*

## Background

Cardiovascular disease is the leading cause of death in patients with Duchenne muscular dystrophy (DMD) [1–3]—a fatal X-linked genetic disorder characterized by progressive skeletal, respiratory, and cardiac muscle weakness. DMD affects 15.9 to 19.5 per 100,000 live births, making it the most common muscular dystrophy in kids and fatal genetic disorder. Advancements in respiratory clinical management has enabled boys with DMD to live longer, thereby revealing the cardiac complications that arise. DMD is associated with a variable onset of pediatric cardiomyopathy and heart failure by early adulthood [1, 3]. Clinical evidence of cardiac dysfunction is frequently limited to imaging findings until severe or end-stage cardiomyopathic change has occurred since symptom recognition is difficult in non-ambulatory patients. Consequently, sensitive imaging methods are helpful to identify early cardiac involvement in this high-risk population.

Ongoing efforts to develop DMD-specific therapies may prolong life as they delay the onset of cardiomyopathy in this patient population. However, evaluating the cardiovascular response to novel therapies proves challenging due to the lack of validated cardiac imaging biomarkers for DMD disease progression. Echocardiography and cine cardiovascular magnetic resonance (CMR) imaging enables quantitative estimates of global left ventricular (LV) function including systolic and diastolic volumes, myocardial strain, and LV ejection fraction (LVEF). These metrics, however, are only sensitive to overt functional changes and do not provide insight to microstructural remodeling that may contribute to subclinical changes in heart health, fomenting myocardial fibrosis, and overall disease progression.

Cardiac microstructural remodeling in DMD has been identified on pathology as progressive fibrofatty infiltration in the sub-epicardium of the LV free wall, most notably at the base of the heart [4, 5]. This level of myocardial remodeling can also be detected using the conventional late gadolinium enhancement (LGE), which is the current gold standard for detecting myocardial tissue remodeling. LGE imaging has utility for detecting focal replacement fibrosis, but it is often a late finding (mean onset observed at 15.2 $\pm$ 5.1 years [6]) and it underestimates the extent of cardiac involvement because it does not quantify the level of diffuse fibrosis. Diffuse fibrosis, however, is an earlier indicator of cardiac involvement in this population [6, 7]. Due to its need for contrast administration, LGE imaging may be considered invasive and make it challenging for pediatric patients to endure. Increasingly, there exists interest in non-contrast CMR methods to evaluate myocardial remodeling. Importantly, a biomarker capable of detecting early myocardial remodeling prior to LGE can significantly improve the care of boys with DMD. This is especially important given the certainty with which boys will develop cardiac involvement, but the uncertainty associated with the timing of the onset.

Emerging CMR biomarkers have shown promise in quantifying myocardial remodeling by T_1_-mapping, whereby tissue-specific changes can be monitored over time in several cardiac pathologies [8]. To date, T_1_ mapping studies in DMD have been reported predominantly at 1.5 T and have demonstrated the ability of native (pre-contrast) LV myocardial T_1_ measurements to distinguish between healthy hearts and hearts with positive LGE (LGE+) and negative LGE (LGE−) findings in boys with DMD [4, 8–11]. One study revealed shortened LV myocardial post-contrast T_1_ as another measure of fibrosis that may be detected prior to LGE+ findings in DMD [11]. Additionally, from pre- and post-contrast T_1_ measurements (and if the patient’s hematocrit is measured), the extracellular volume (ECV) fraction can be calculated and used to quantify diffuse fibrosis [12].

The clinical use of 3T CMR continues to increase due to the wide installation base and owing to its many advantages: higher signal-to-noise (SNR) and contrast-to-noise ratio (CNR), faster acquisition times, and more effective functional and microstructural imaging [17, 18]. However, no reports are currently available for native T_1_ and ECV estimates in pediatric patients with DMD at 3T. Herein we aim to use 3T CMR: (1) to characterize global and regional myocardial native T_1_ differences between boys with DMD and healthy controls; (2) to report global and regional myocardial post-contrast T_1_ values and myocardial ECV estimates in boys with DMD; and (3) to identify LV T_1_-mapping biomarkers capable of distinguishing between healthy controls and boys with DMD and detecting LGE status in DMD.

## Methods

### Study enrollment

This two-center prospective CMR study was approved by both Institutional Review Boards and completed between January 2017 and January 2020. We obtained parental consent and child assent for all study participants under the age of 18 years. Boys with DMD were recruited from one of two children’s hospitals on a referral basis from two counties with large urban populations. DMD diagnosis was confirmed by genetic testing to identify the presence of a dystrophin mutation. Boys with DMD did not require respiratory support and were not enrolled in any therapeutic clinical trial at the time of the study. Healthy participants were recruited from the surrounding communities and had no history of cardiovascular disease. Site-A enrolled 13 healthy controls and 21 boys with DMD. Site-B enrolled 7 healthy controls and 7 boys with DMD. In total, 28 boys with DMD and 20 sex-matched healthy controls were enrolled. Table 1 displays the demographic information for the two groups and a summary of medications taken by boys with DMD at the time of the CMR exam.

**Table 1:** Demographics of healthy controls and boys with DMD

	Healthy Controls N = 20	DMD N = 28
Age (years)	13 (4.0) range (9–21)	13 (4.5) range (9–21)
Male (%)	100%	100%
Height* (cm)	165 (21)	135 (26)
Weight (kg)	51 (15)	50 (28)
BMI* (kg/m^2^)	18.2 (3.4)	25.5 (10.2)
BSA* (m^2^)	1.5 (0.3)	1.4 (0.4)
Heart rate* (bpm)	69 (30)	84 (24)
Ambulatory (%)	20 (100%)	3 (10.7%)
Ventilatory Support (%)	0%	0%
*Race*		
Caucasian	15	15
African American	1	1
Asian	2	5
Other	2	5
Mixed	0	2
*Ethnicity*		
Hispanic/Latino	7	10
*Medications at CMR*		
ACEi	0	21 (75%)
ARB	0	4 (14%)
$\beta$-blocker	0	7 (25%)
Corticosteroids	0	18 (64%)
Diuretic	0	16 (57%)

*ACEi*, Angiotensin Converting Enzyme Inhibitor; *ARB*, Angiotensin Receptor Blocker; *BMI,* body mass index; *BSA,* body surface area

^*^p ≤ 0.05; Results are presented as median (IQR)

### Cardiovascular magnetic resonance

After providing informed consent, all 45 participants underwent 3 T CMR (Skyra, Siemens Healthineers, Erlangen, Germany) at each site, using identical software, coils, and scan protocol. The CMR exam included standard functional imaging using a high spatial and temporal resolution, free-breathing retrospectively binned balanced steady state free precession (bSSFP) cine sequence [19, 20] with the following acquisition parameters: 40º flip angle, 6/8 partial Fourier and rate-4 parallel imaging, matrix size 192 × 144, pixel size 1.9 mm × 1.9 mm, slice thickness 8 mm, bandwidth (BW) 930 Hz/Px, TE/TR 1.2 ms/2.4 ms echo spacing, and a temporal resolution of 64.4 ms. Breath-held bSSFP cine was used in six study participants when the free-breathing sequence was unavailable. Parameters for this acquisition were: 58º flip angle, rate-3 parallel imaging, matrix size 256 × 192, pixel size 1.6 mm × 1.6 mm, slice thickness 6 mm, BW 977 Hz/Px, TE/TR 1.4 ms/3.3 ms echo spacing, and a temporal resolution of 32.5 ms. All cine imaging spanned the entire LV from base to apex using short-axis slices.

Myocardial T_1_ measurements were acquired using a modified Look-Locker inversion recovery (MOLLI) sequence with MOtion Correction (MOCO) [11] in a single mid-ventricular short-axis (SAx) slice and was performed with electrocardiographic (ECG)-gating and breath holding. Pre- and post-contrast T_1_ mapping was acquired with a 5(3 s)3 and a 4(1 s)3(1 s)2 MOLLI scheme, respectively. Typical native T_1_ imaging parameters were: non-selective inversion pulse, bSSFP single shot  readout with a 20º excitation flip angle, 7/8 partial Fourier and rate-2 parallel imaging, matrix size 192 × 132, pixel size 1.9 mm × 1.9 mm, slice thickness 8 mm, BW 1085 Hz/Px, minimum inversion time (TI) of 100 ms and incremented by 80 ms, TE/TR 1.01 ms/2.44 ms echo spacing. Typical post-contrast T_1_ imaging parameters were: non-selective inversion pulse, bSSFP single shot read out with a 20º excitation flip angle, 7/8 partial Fourier and rate-2 parallel imaging, matrix size 192 × 164, pixel size 1.9 mm × 1.9 mm, slice thickness 8 mm, BW 1085 Hz/Px, minimum TI of 100 ms with 80 ms increments, TE/TR 1.01 ms/2.44 ms echo spacing. For heart rates greater than 90/min, the matrix size was decreased to 192 × 128 to mitigate any heart rate biases. Two subjects were inadvertently scanned with the 4(1 s)3(1 s)2 MOLLI scheme for their pre-contrast scan. We have found that this error does not significantly impact the group pre-contrast results. We computed the percent error between each such subject’s pre-contrast T_1_ and the group pre-contrast mean and found the measurements themselves do not vary significantly from the group mean (percent error < 5% for both measurements).

Gadobenate dimeglumine contrast (Gd-BOPTA, MultiHance, Bracco Diagnostics, Milan, Italy) was administered either by hand or computer controlled injection only to boys with DMD. Eight minutes following contrast administration, LGE imaging with a free breathing motion corrected phase sensitive inversion recovery (PSIR) sequence [21] was acquired in the short axis (SAx) view spanning base to apex using the following parameters: 20º flip angle, rate-2 parallel imaging, matrix size 192 × 120, pixel size 1.4 mm × 1.4 mm, slice thickness 6 mm, BW 977 Hz/Px, TE/TR 2.01 ms/2.83 ms echo spacing, and a temporal resolution of 35.1 ms. Vertical long axis (VLA) and horizontal long axis (HLA) views were also acquired. Approximately 18 min (18 $\pm$ 6.1 min) after contrast injection, post-contrast T_1_ mapping was performed at slice locations matched to the pre-contrast acquisition. The MOCO T_1_ (pre- and post-contrast) maps were generated on the scanner and later used for calculating ECV maps. All boys with DMD provided a blood sample on the day of the CMR exam for measurement of hematocrit to be used in calculating the subject specific ECV.

### Post-processing and analysis

Two expert clinicians (either PR or AP) contoured the images, individually at their respective site, using Circle CVI42 (Circle Cardiovascular Imaging Inc., Calgary, Canada) and Medis (Medis Cardiovascular Imaging, Leiden, the Netherlands). They calculated and analyzed the following functional metrics: LV end systolic volume (LVESV) and end diastolic volume (LVEDV), ejection fraction (LVEF), and LV mass (LVM). Parameters were indexed by body surface area (BSA) to derive LVESVI, LVEDVI, and LVMI. A normal LVEF was defined as LVEF ≥ 55% [22]. Additionally, the clinicians noted the presence or absence of LGE and indicated the number of affected segments according to the American Heart Association (AHA) 17-segment model [23]. A patient with LGE presence in at least one myocardial segment was considered to be LGE positive (LGE +). If no enhancement was observed, then the subject was identified as LGE negative (LGE−). 26 LGE exams were analyzed by both clinicians with two exams excluded due to poor image quality.

Pre- and post-contrast T_1_ maps were registered using a combination of two-dimensional rigid and affine image registration techniques using MATLAB (MathWorks, Natick, Massachusetts, USA) software, then combined with each DMD participant’s hematocrit to calculate an ECV map [9]. A region of interest (ROI) encompassing the LV myocardium, and two additional ROIs including a septal and lateral wall segment, were manually selected and analyzed. From each ROI, summary statistics including within-slice standard deviation (SD) were extracted for the global, septal, and lateral LV myocardial regions (Additional file 1: Figure S1). An agreement analysis of the T_1_ mapping measurements between the two sites was performed to ensure the applicability of both DMD and healthy control data in the group-wise comparisons. Site-specific measurements were compared using a Wilcoxon-rank sum test.

Demographics for the boys with DMD and healthy controls were compared using a Wilcoxon rank-sum test. Following skewness and kurtosis tests for normality, group-wise comparisons of segmental and global myocardial pre-contrast T_1_ were performed with a Wilcoxon rank-sum test between boys with DMD and healthy controls. The segmental and global myocardial post-contrast T_1_ and ECV data were compared for two DMD sub-groups (LGE + vs. LGE-). Furthermore, the within-slice standard deviation (SD) for pre-contrast and post-contrast T_1_ and ECV was evaluated in an effort to characterize differences in myocardial tissue heterogeneity between boys with DMD and healthy controls, and also between the two DMD sub-groups. After post hoc correction for multiple comparisons, a *p*-Value < 0.05 was considered significant. Due to the varied progression of DMD within this patient cohort and non-normal distribution of the CMR measurements, data is reported as median and interquartile range (IQR). A linear-regression analysis was used to identify initial correlations between T_1_ metrics and LV function in boys with DMD and healthy controls. R^2^ and p-Values are reported. Multiple-regression analysis was then used to test for correlations between T_1_-mapping (pre- and post-contrast T_1_, and ECV) measured from lateral wall segments, and global functional metrics (LVEF, LVEDVI, LVESVI, LVMI), and Age, BMI, and heart rate covariates. A binomial logistic regression classifier was analyzed using Receiver Operating Characteristic (ROC) analysis for each measured biomarker in the following distinguishing tasks: (1) healthy controls vs. DMD; (2) healthy controls vs. LGE− boys with DMD; and (3) LGE− vs. LGE + boys with DMD. Results are displayed by ROC curves and the area under the ROC curve (AUC) is reported. ROC curves and AUCs are compared for each individual biomarker. A combination (native T_1_ and LVEF) is used to evaluate the discriminatory power of non-contrast biomarkers. All statistical analyses were performed in MATLAB (Mathworks).

## Results

### Demographics

We found four significant demographic differences between the two groups: boys with DMD had faster heart rates and were shorter, resulting in larger BMI and smaller BSA values compared to healthy controls (Table 1).

### LV volume and function

Boys with DMD had significantly reduced LVEF [49.5 (11.3) % vs 55.9 (5.8) %); *p-*value = 0.003] and lower LVMi [35.6 (9.8) g/m^2^ vs 38.4 (7.8) g/m^2^; *p*-value = 0.04]. Among boys with DMD, 17 out of the 28 (61%) presented with reduced LVEF. There were no significant differences in LVEDVI, and LVESVIi between the two groups, but boys with DMD had a smaller LVEDVI and larger LVESVI compared to healthy controls. Indices of LV function are displayed in Table 2.

**Table 2:** Metrics of left ventricular function from standard CMR

	Healthy Control N = 20	DMD N = 28	*p*-Value	DMD LGE− N = 17	*p*-value	DMD LGE + N =9	*p*-Value
LVEF (%)	55.9 (5.8)	49.5 (11.3)	0.003	55.2 (10.9)^#^	0.20	44.8 (10.7)	< 0.001
LVEDVI (ml/m^2^)	87.7 (15.2)	82.8 (27.9)	0.10	68.2 (26.8)^#^	0.02	91.8 (39.6)	0.81
LVESVI (ml/m^2^)	38.5 (8.5)	38.7 (14.5)	0.89	36.4 (9.7)^#^	0.02	45.5 (28.7)	0.03
LVMI (g/m^2^)	38.4 (7.8)	35.6 (9.8)	0.04	32.4 (8.4)	0.02	39.5 (8.7)	0.94

All subgroups compared to healthy controls. p-value ≤ 0.05 is significant

*LVEF,* left ventricular ejection fraction; *LVEDVI* left ventricular end-diastolic volume; *LVESVI* left ventricular end-systolic volume; *LVMI* left ventricular mass index; *LGE-, *late gadolinium enhancement negative; *LGE*+, late gadolinium enhancement positive

^#^p ≤ 0.05 comparison between LGE − and LGE + boys

### Late gadolinium enhancement

**Fig. 1:**
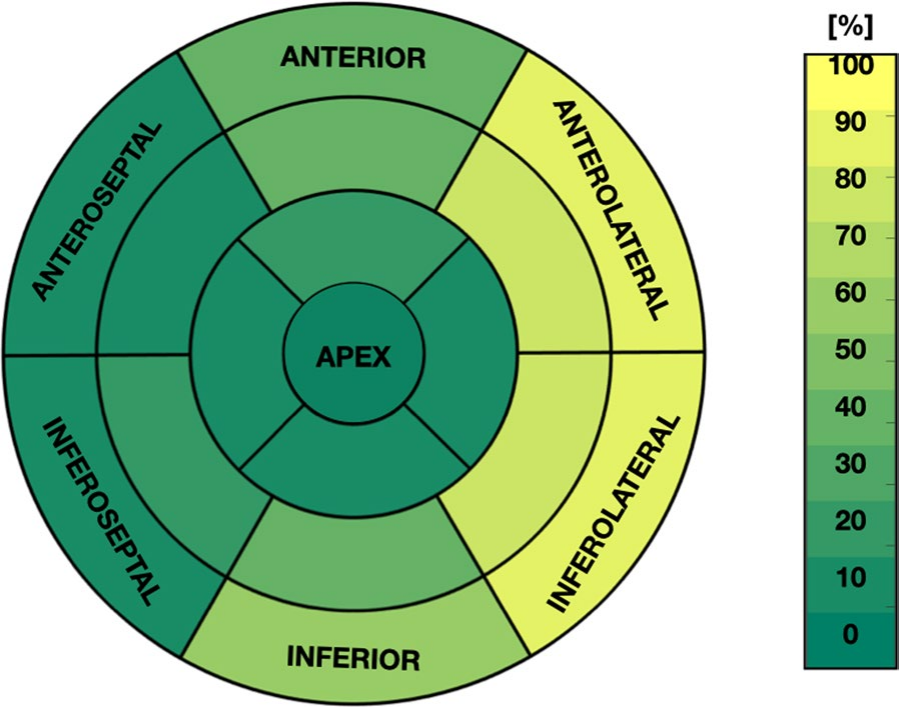
Affected Segments for LGE+ Boys with DMD. LGE+ segment distribution for LGE+ boys with DMD (N = 9). The majority of the affected segments are found in the anterolateral and inferolateral LV wall, whereas the septum is less frequently affected

Nine (32%) of the DMD boys were LGE+ with at least one myocardial segment. Figure 1 shows the distribution of LGE+ segments for all nine LGE+ boys with DMD. Furthermore, all LGE+ boys had enhancement present in the mid-ventricular slice used for T_1_ mapping. One significant demographic difference was observed in this group: LGE+ DMD boys had a lower heart rate [73.5 (14.5) bpm vs 96 (28.8) bpm; *p* = 0.01] compared to LGE− patients. For all LGE+ boys with DMD, the clinicians observed enhancement present at the lateral LV wall, but in two boys, enhancement was also present at the septal wall. LGE+ patients with DMD had a significantly larger LVEDVi [91.8 (39.6) g/m^2^ vs. 68.2 (26.8) g/m^2^; *p* = 0.02] and LVESVI [45.5 (28.7) g/m^2^ vs 36.4 (9.7) g/m^2^; *p* = 0.001] and lower LVEF than LGE− patients with DMD [44.8 (10.7) % vs 55.2 (10.9) %; *p* = 0.005]*.*

### T_1_ mapping between sites

**Fig. 2:**
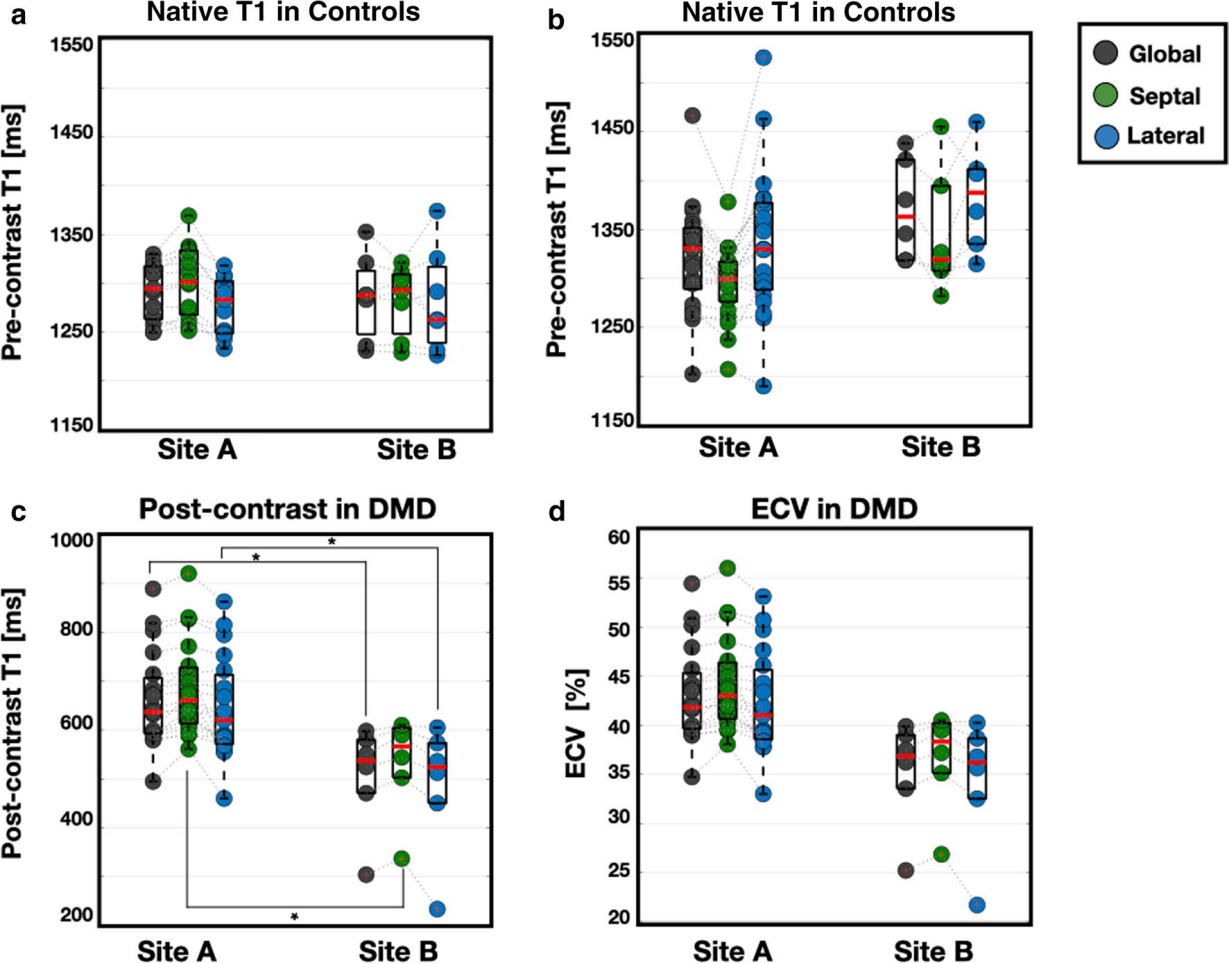
Box plots of regional (global, septal, and lateral) native T_1_ (**a**, **b**), post-contrast T_1_ (**c**), and ECV (**d**) for Site-A and Site-B. No significant differences observed between the two sites for pre-contrast T_1_ measurements in healthy controls (**a**) and pre-contrast T_1_ (**b**) and ECV (**d**) in boys with DMD. Boys with DMD scanned at Site-A had significantly increased post-contrast T_1_ (**c**) measurements compared to boys scanned at Site-B

We performed an agreement analysis between the two sites (i.e. Site-A and Site-B) and found no statistically significant differences in the measured native T_1_ values between healthy controls (Fig. 2a) and boys with DMD (Fig. 2b). Similarly, no significant difference was observed in ECV measurements (Fig. 2d) between the two sites. Post-contrast T_1_ measurements between the two sites, however, were found significantly different for all three regions of interest (global, septal, and lateral) as seen in Fig. 2c. Post-contrast measurements from Site-B were significantly lower than measurements from Site-A. To better understand these site specific differences, we further assessed the pre- and post-contrast blood pool T_1_ measurements from all controls and boys with DMD, the blood hematocrit, and the average time after contrast injection for boys with DMD (Table 3). Significant differences were found only in the post-contrast blood pool measurements [406 (197) ms vs. 324 (100) ms; *p* = 0.03] between Site-A and Site-B and in the blood hematocrit measurements [44 (2.4) % vs. 40 (3.0) %; *p* = 0.01], respectively.

**Table 3:** Site-specific T1 measurements

Parameter	Site A	Site B	p-Value
Control pre-contrast blood pool T1 (ms)	1881 (62)	1801 (79)	0.10
DMD pre-contrast blood pool T1 (ms)	1816 (137)	1881 (105)	0.10
DMD post-contrast blood pool T1 (ms)	406 (197)	324 (100)	0.03
DMD blood hematocrit (%)	44 (2.4)	40 ( 3.0)	0.01
Average time after contrast injection (min)	17 (7.9)	18 (9.3)	0.77

### T_1_ mapping and extracellular volume in DMD

**Fig. 3:**
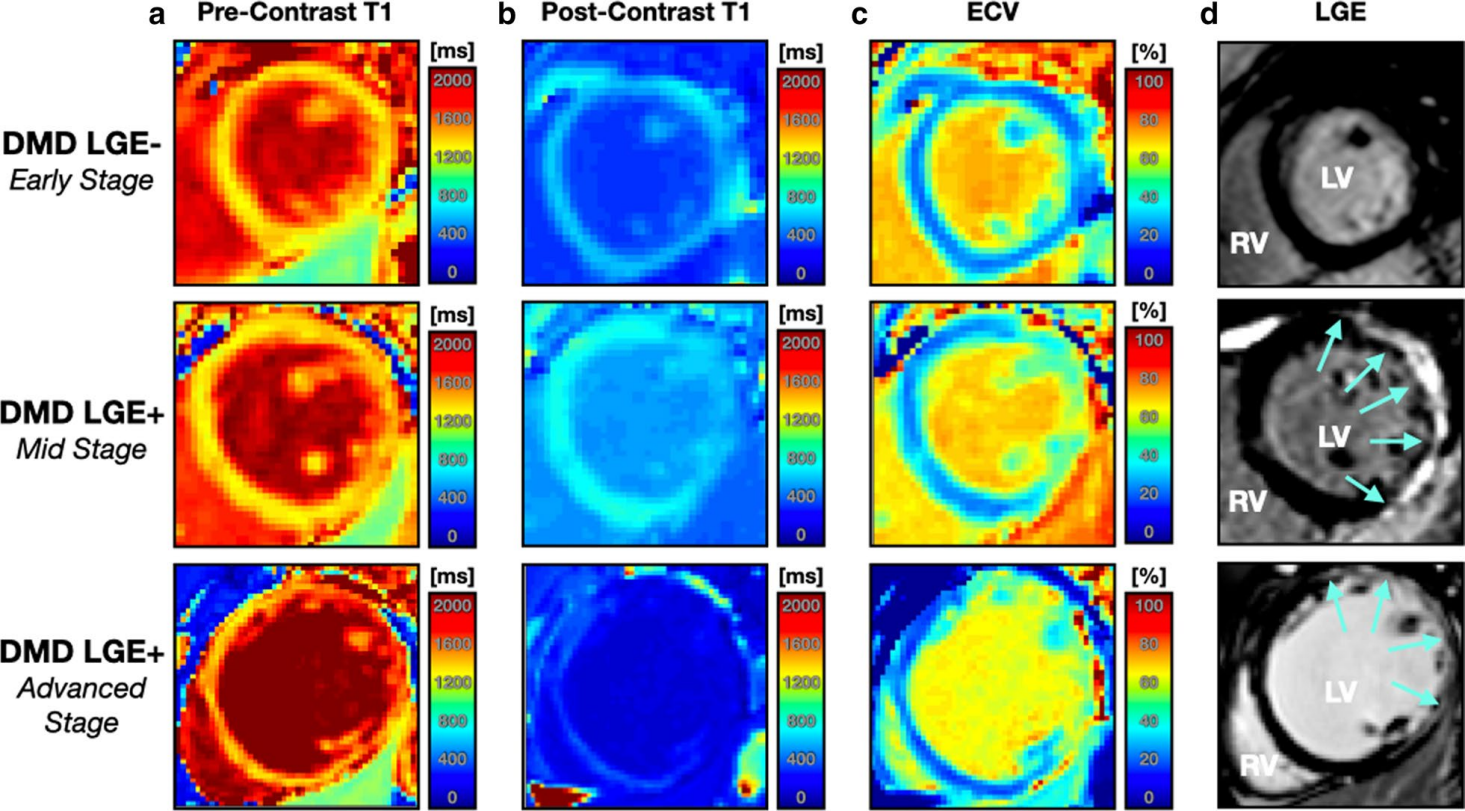
Example mid-ventricular short axis (SAx) (pre-contrast) native T_1_ and post-contrast T_1_ maps (columns **a**, **b**), extracellular volume fraction (ECV) maps (column **c**), and corresponding late gadolinium enhancement (LGE) images (column **d**) with areas of enhancement (arrows). Example maps and images are shown for an LGE− boy with DMD at an early stage of cardiac involvement (first row), for an LGE + boy with DMD at mid stage cardiac involvement (middle row), and for an LGE+ boys with DMD with advanced cardiac involvement. *LV* left ventricle; *RV* right ventricle

All native T_1_ maps were analyzed for the healthy controls. Within the DMD cohort, three patients were unable to complete the entire CMR exam resulting in 27 (96%) native T_1_, and 25 (89%) post-contrast T_1_ and ECV maps analyzed. Figure 3 displays example pre-contrast and post-contrast T_1_ maps (columns A-B), ECV maps (column C), and LGE images (column D) for three boys with DMD at varying stages of cardiac involvement. Guided by previous studies [24, 25], boys with DMD for this study were defined to be in the early stages of cardiac involvement if they were LGE− with normal LVEF. Mid-stage patients were defined more broadly: (1) either a patient was LGE− with reduced LVEF or; (2) also if the patient was LGE+ with normal LVEF. Advanced stage cardiac involvement was defined as LGE+ with reduced LVEF and visibly dilated LV. Tables 4 and 5 summarize the T_1_ mapping results.

**Table 4:** Summary T_1_ mapping and ECV differences between DMD patients and healthy controls

	Healthy Control N = 20	DMD N = 28	LGE− N = 17	LGE+ N = 9
*Native/pre-contrast T*_*1*_*(ms)*				
Global	1289 (56)	1332 (60)*	1315 (57)^#^	1350 (53)*
Septal	1300 (55)	1308 (40)	1299 (38)	1318 (54)
Lateral	1277 (58)	1348 (86)*	1322 (68)*^#^	1380 (71)*
*Post-contrast T*_*1*_*(ms)*				
Global		598 (96)	635 (126^)#^	565 (113)^#^
Septal		639 (112)	643 (113)	591 (125)
Lateral		591 (128)	613 (134)^#^	542 (93)^#^
*ECV (%)*				
Global		30 (4)	28 (4)	32 (8)
Septal		27 (3)	27 (4)	27 (4)
Lateral		30 (8)	29 (6)^#^	38 (7)^#^

All subgroups compared to healthy controls

^*^p-value ≤ 0.05 is significant

^#^p-value ≤ 0.05 comparison between LGE− and LGE + patients

**Table 5:** Summary within-slice standard deviation differences between boys with DMD and healthy controls

	Control N = 20	DMD N = 28	LGE− N = 17	LGE+ N = 9
*Native/pre-contrast T*_*1*_* (ms)*				
Global	74 (27)	100 (57)*	100 (37)*	104 (61)*
Septal	63 (31)	87 (31)	81 (25)*	92 (46)
Lateral	67 (27)	98 (46)*	91 (29)*	98 (47)*
*Post-contrast T*_*1*_* (ms)*				
Global		56 (24)	50 (14)^#^	81 (34)
Septal		39 (18)	35 (12^)#^	52 (14)
Lateral		57 (35)	50 (17^)#^	80 (37)
*ECV (%)*				
Global		7 (3)	6 (2)^#^	11 (7)
Septal		5 (2)	5 (2)	6 (1)
Lateral		5 (4)	6 (3)^#^	11 (8)

All subgroups compared to healthy controls

^*^p-value ≤ 0.05 is significant

^#^p-value ≤ 0.05 comparison between LGE− and LGE + patients

**Fig. 4:**
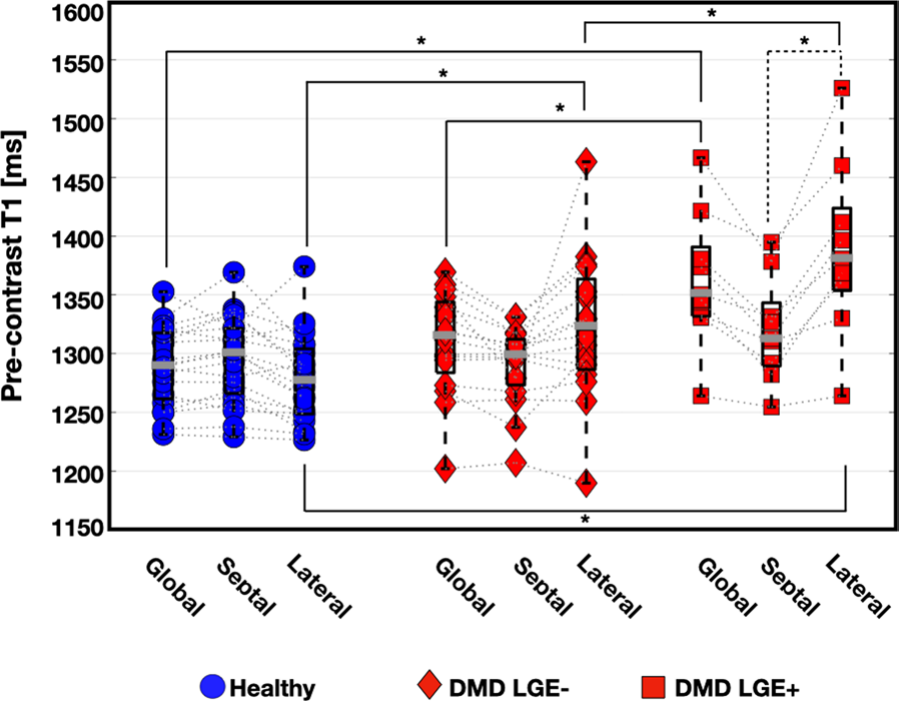
Box plots of regional pre-contrast T_1_ values in the DMD and healthy control groups. While the boys in both DMD subgroups exhibited elevated pre-contrast T_1_ compared to healthy controls, these differences did not reach statistical significance at the septal level. Global myocardial T_1_ values in the both DMD subgroups were significantly increased compared to healthy controls. In the lateral myocardium, both DMD subgroups had a significantly elevated pre-contrast T_1_ compared to healthy controls. In the LGE+ DMD subgroup, the lateral myocardial region exhibited a significantly elevated pre-contrast T_1_ compared to the septal region

Compared to healthy controls, DMD subjects had significantly increased global myocardial native T_1_ [1289 (56) ms vs. 1332 (60) ms; *p* = 0.004; Fig. 4] and significantly increased within-slice pre-contrast T_1_ SD [74 (27) ms vs.100 (57) ms; *p* = 0.001; Table 5]. In the lateral wall, native T_1_ [1348 (86) ms vs. 1277 (58) ms vs.; *p* = 0.001] and within-slice native T_1_ SD [98 (46) ms vs. 67 (27) ms; *p* = 0.001; Table 4] remained significantly increased in boys with DMD compared to healthy controls. The septal myocardium showed no significant differences in native T_1_ [1300 (55) ms vs 1299 (38) ms; *p* = 0.64; Fig. 4], nor within-slice SD [63 (31) ms vs 87 (31) ms; *p* = 0.06; Table 5] between healthy and DMD boys, respectively.

LGE+ boys with DMD had significantly increased native T_1_ [1350 (53) ms vs 1315 (57) ms; *p* = 0.05] and lateral [1380 (71) ms vs 1322 (68) ms; *p* = 0.04] myocardial native T_1_ compared to LGE− boys with DMD respectively (Table 4). No significant septal myocardial nor within-slice SD differences were observed between LGE− and LGE+ boys with DMD (Table 5). Lateral myocardial native T_1_ was significantly increased in LGE− patients compared to healthy controls [1322 (68) ms vs 1277 (58) ms; *p* = 0.02; Fig. 4]. Figure 4 also shows within-group regional T_1_ differences for the three groups (controls, LGE+ DMD, and LGE− DMD). Compared to the lateral myocardium, the septal region had significantly lower pre-contrast T_1_ values in both DMD subgroups. No significant within-group regional differences were observed in the healthy myocardium. LGE− boys with DMD with normal LVEF were compared against LGE− boys with reduced LVEF, but no significant differences were observed in native T_1_ measurements [1315 (87) ms vs. 1308 (24) ms; p = 0.93], respectively.

**Fig. 5:**
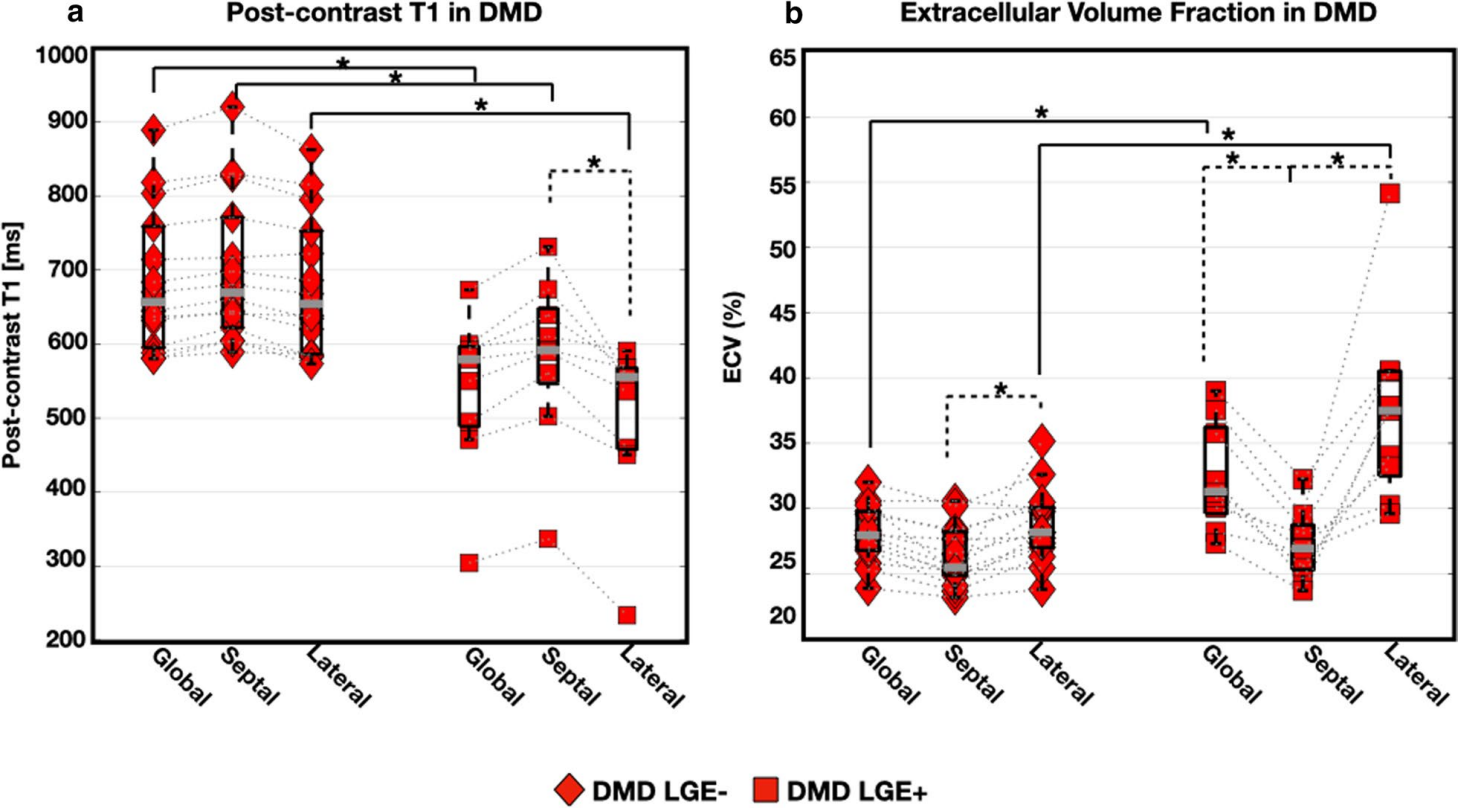
Paired box plots of regional post-contrast T_1_ (**a**) and ECV (**b**) in the DMD cohort. A significant decrease in post-contrast T_1_ is observed in all myocardial regions for LGE+ boys with DMD compared to LGE− boys with DMD. In LGE + boys with DMD, lateral post-contrast T_1_ is significantly decreased from the septal region. Global and lateral myocardial ECV is significantly increased in LGE+ boys with DMD compared to LGE− boys. Furthermore, septal ECV is significantly decreased from lateral ECV in both LGE+ and LGE− DMD subgroups

Figure 5 displays regional post-contrast T_1_ and ECV measurements from the cohort of boys with DMD. The following septal, and lateral myocardial post-contrast T_1_ values were observed: 639 (112) ms, and 591 (128) ms, respectively. A pattern of decreased post-contrast T_1_ in the lateral wall compared to measurements in the septal wall was observed, but this difference only reached significance in LGE+ boys with DMD [542 (93) ms vs. 613(134); *p* ≤ 0.05]. This pattern of shortened post-contrast T_1_ in lateral myocardium is also clearly depicted in Fig. 3.

ECV measurements demonstrated significant differences between the septal and lateral myocardium in patients with DMD [27 (3) % vs. 30 (8) %; *p* = 0.001; Fig. 5]. This result is consistent with diffuse fibrosis and extracellular expansion occurring in the DMD disease process [4]. LGE + boys had a significantly increased lateral myocardial ECV [38 (7) % vs. 29 (6) %, *p* = 0.001; Table 4 compared to LGE− boys. However, at the septal level, no significance was reached for ECV [27 (4) % vs. 27 (4) %, *p* = 0.73; Table 4] comparison between LGE+ and LGE− boys with DMD. Similar to pre-contrast T_1_, no significant differences were observed in post-contrast T_1_ [641 (103) ms vs. 610 (178) ms; p = 0.49] or ECV [28 (4) % vs. 27 (3) %; p = 0.57] measurements between LGE− boys with normal LVEF and LGE− boys with reduced LVEF.

### T_1_ mapping, extracellular volume, and LV function

Significant correlations between T_1_ mapping and metrics of LV function in DMD patients were observed. In the LGE+ group, pre-contrast T_1_ and LVEDVI [R^2^ = 0.68, p = 0.01], native T_1_ and LVMI [R^2^ = 0.56, p = 0.02], and post-contrast T_1_ and LVEF [R2 = 0.53, p = 0.03] were significantly correlated. In LGE− boys with DMD, a significant correlation was observed between post-contrast T_1_ and LVEF only [R^2^ = 0.49, p = 5.4 × 10^–3^]. No T_1_ and functional metrics were correlated in healthy controls. Additional file 2: Figure S2 A-D illustrates the significant correlations mentioned above.

**Fig. 6:**
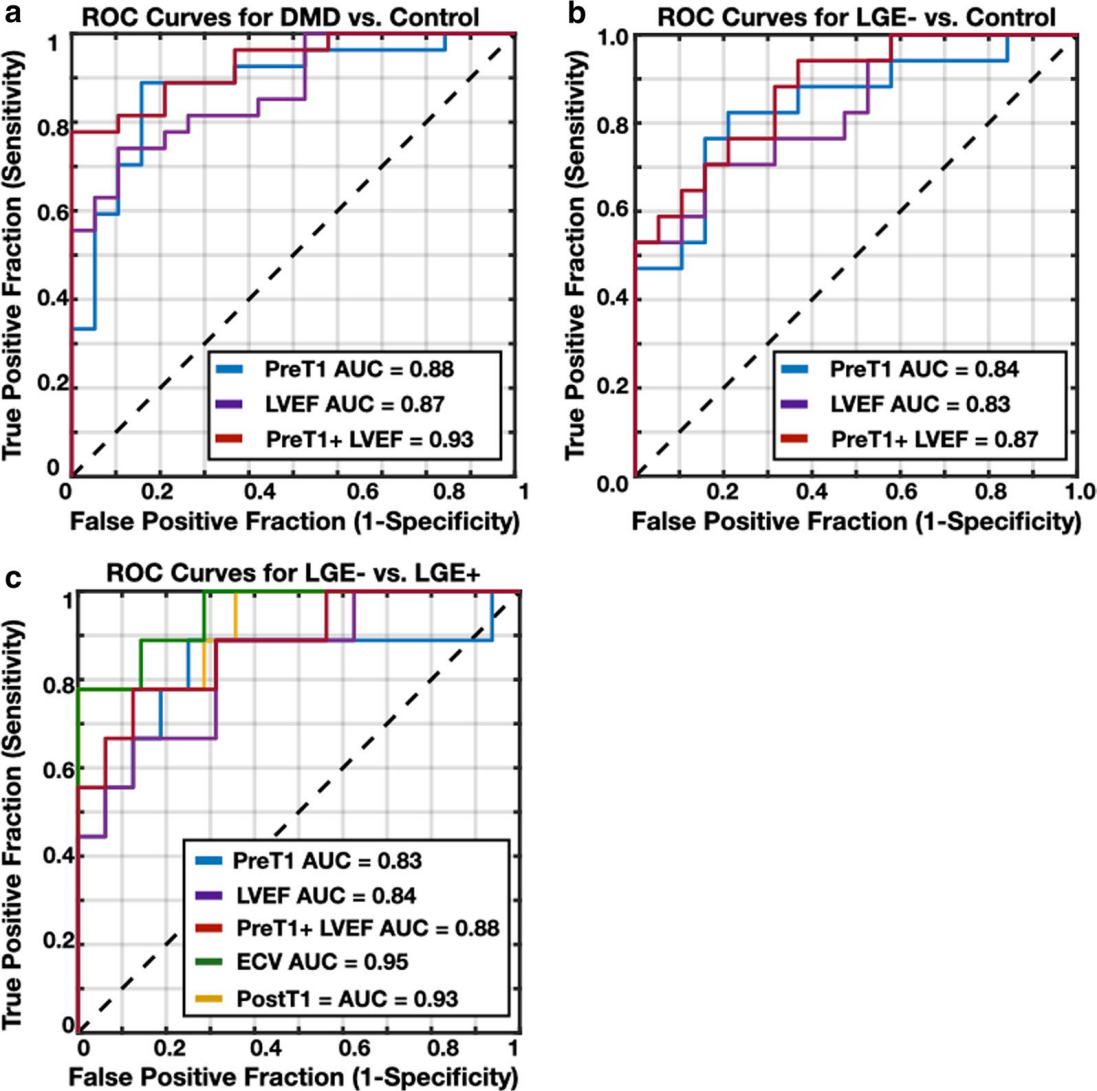
Receiver operating characteristic (ROC) curves for individual lateral wall native and post-contrast T_1_ mapping biomarker measurements and LVEF from a binomial logistic regression classifier in the task of distinguishing between boys with DMD from healthy controls (**a**), LGE− boys with DMD from healthy controls (**b**), and LGE− from LGE + boys with DMD (**c**). In all classification tasks, T_1_-mapping biomarkers outperform a conventional biomarker, LVEF. When non-contrast biomarkers (native T_1_ and LVEF) are combined, the classification model improves for all three classification tasks, compared to the performance of each biomarker alone. ECV is the best performing biomarker in the task of predicting LGE status

The ROC evaluation revealed that a binomial logistic regression classifier using each biomarker in combination with the age, BMI, heart rate, and LVMI as features in all classification tasks resulted in a better model performance than each biomarker alone. Furthermore, the ROC analysis illustrated that all T_1_-mapping biomarkers and LVEF are significant predictors of DMD and LGE status (AUC > 0.50); Fig. 6 displays the ROC curves for all the classification tasks. In the task of distinguishing between boys with DMD and healthy controls, native T_1_ was comparable to LVEF (AUC = 0.88 vs. AUC = 0.87), but the combination of pre-contrast T_1_ and LVEF yielded the best performance (AUC = 0.93). Figure 6b displays the same behavior in the LGE− vs. LGE + boys with DMD using native T_1_, LVEF, and the combination of the two biomarkers (AUC = 0.84 vs. AUC = 0.83 vs. AUC = 0.87), respectively. In the task of predicting LGE status, ECV (AUC = 0.95) outperformed pre- and post-contrast T_1_ (AUC = 0.83, AUC = 0.93), and LVEF (AUC = 0.84). The combination of native T_1_ and LVEF (AUC = 0.88) again, performed better in the task of distinguishing between LGE + vs. LGE− boys with DMD compared to each biomarker performing individually.

## Discussion

This study used T_1_ mapping to define the cardiac microstructural differences found between pediatric patients with DMD and healthy, sex- and age-matched controls at 3T. To our knowledge, this is the first study to evaluate T_1_ mapping in a pediatric DMD study population at 3T. Therefore, these data help to establish reference values for both boys with DMD and healthy controls at 3T. Additionally, the study presented here is the first to investigate a classification model for identifying T_1_ mapping differences between boys with DMD and healthy controls and for predicting the presence of pathology associated with LGE status in DMD without requiring contrast. This study further provides evidence to support non-contrast exams in pediatric DMD patients specifically, and can be expanded to investigate T_1_ mapping in other cardiomyopathies, particularly in settings when the use of contrast might be contraindicated.

As expected, the 3T native T_1_ values reported from this study are elevated relative to previously reported 1.5T pre-contrast T_1_ values [6, 14–16, 26]. While elevated, the reported increase in pre-contrast T_1_ in boys with DMD compared to healthy controls is consistent with previously published studies at 1.5 T [15, 16, 26]. Taken together these findings further confirm the sensitivity of T_1_ mapping for assessing myocardial abnormalities in this population.

Soslow et al*.* reported increased native T_1_ at 1.5T in DMD patients (N = 31; age 13.4 ± 4.7 years; all males) compared to healthy controls (N = 11; age 24.5 ± 3.9; all males) [1045 ms vs 988 ms, p = 0.001] [15]. They also demonstrated that this trend remained for LGE− DMD patients with normal LVEF compared to healthy controls. Olivieri et al*.* demonstrated that DMD boys (N = 20, age 14.4 ± 4 years) also had significantly elevated native T_1_ values (*p* < 0.05) compared to healthy sex-matched controls (N = 16; age 16.1 ± 2.2 years) using both SASHA and MOLLI techniques. Furthermore, when compared to ECV, pre-contrast T_1_ demonstrated a 50% increase in the ability to distinguish healthy controls from LGE− boys with DMD, and also from LGE+ boys with DMD. Another study at 1.5T by Pavnosky et al. assessed the myocardium of a DMD patient population and also noted a significantly increased native T_1_ (*p* < 0.05) in LGE+ and LGE− DMD groups compared to healthy controls.

The native T_1_ differences observed in this study (and the above mentioned studies) between DMD patients and healthy controls are consistent with known pathological findings such as fibrosis resulting from extracellular matrix expansion in DMD muscle [6, 9, 27]. Importantly, these changes are detectable even in DMD patients who present with negative findings on LGE exams and therefore provides an earlier indication of cardiac involvement. The success of using pre-contrast T_1_ to detect other pathologies [13] coupled with on-going concerns regarding the use of gadolinium-based contrast agents [28, 29] further motivates the clinical use of pre-contrast T_1_. As shown by the agreement analysis between Site-A and Site-B, pre-contrast T_1_ is also more consistent, which makes it better for direct comparisons across sites. Importantly, native T_1_ could be used as an early, non-invasive surrogate biomarker for monitoring subclinical cardiac microstructural changes in DMD, thereby enabling earlier and more patient-specific treatment options.

The ECV values reported herein are consistent with previously published pediatric studies [15, 16, 26, 30], showing the potential for ECV as both a reproducible and repeatable biomarker invariant to magnetic field strength. Furthermore, the global myocardial ECV of DMD patients from this study [30 ± 5%] was increased compared to that of published healthy controls [24 ± 1% [15]]. Elevated myocardial ECV in DMD subjects compared to healthy controls has been shown by multiple studies [6, 15, 16, 26, 30], thus ECV is promising as a quantitative metric for detecting myocardial microstructural remodeling. Furthermore, this study detected increased ECV in LGE+ patients compared to LGE− patients; a finding also demonstrated by Soslow et al*.* [15] The studies by Olivieri et al*.* [16] and Panovsky et al*.* [26] only predicted the presence of LGE, but did not distinguish between control subjects and LGE− DMD patients. Such dissimilar findings likely arise due to a variety of cohort specific factors, including the dependence of the results upon the stage of disease.

The regional analysis of pre-contrast and post-contrast T_1_ and ECV mapping confirms the disease pattern of fibrosis in the myocardium of boys with DMD. This disease pattern is reported in pathology and imaging studies [27]. Significantly increased native T_1_ and ECV, and significantly decreased post-contrast T_1_ are observed in the lateral wall compared to septal wall of boys with DMD. These findings are consistent with previously published studies noting that affected myocardial segments predominate in the lateral LV [24, 31–33]. These two myocardial regions experience very different loading conditions, owing to the RV pressure acting on the septum, which may underlie the microstructural differences that arise between these regions [33–35].

The regional abnormalities detected by T_1_ mapping are also consistent with the regions in which LGE is present within the DMD myocardium (Fig. 3). While LGE imaging indicates the presence and location of fibrosis, T_1_ mapping provides a quantitative description and enables the assessment of myocardial changes that precede the qualitative observance of LGE. In this study, septal T_1_ measurements could not distinguish between boys with DMD and healthy controls. Consequently, a regional assessment, as carried out in previous studies [15, 16, 26] provides a more meaningful evaluation of myocardial remodeling in the DMD disease process. In fact, to identify the earliest signs of cardiac involvement in boys with DMD, future studies may focus on more basal slices, wherein cardiac involvement appears earlier.

Furthermore, given the pattern of involvement, T_1_ measurements from the septal myocardium may provide a reference (intra-subject control) measure for each individual boy that could provide a way to better monitor microstructural changes over time. Figures 4 and 5 illustrate the regional differences observed in pre-contrast and post-contrast T_1_ and ECV, suggesting that microstructural changes due to DMD predominantly appear in the myocardial lateral wall compared to the septum. In this study, post-contrast T_1_ appears to be a weaker determinant of disease stage and severity, as this data only demonstrates significant differences between the septum and lateral myocardium within the LGE+ DMD group. The observation that regional differences are apparent within boys with DMD provides a valuable internal control that mitigates the problems associated with not having post-contrast T_1_ values in the control group. These findings further motivate continued use of native T_1_ mapping to monitor subclinical changes in the myocardium.

We note significantly greater within-slice standard deviation of native T_1_ in boys with DMD compared to healthy controls in both global and regional myocardial measurements, which could provide a biomarker of myocardial tissue heterogeneity. The T_1_ values obtained are a complex makeup of signal coming from both cardiomyocyte and extracellular matrix components, thus this finding warrants a T_1_ texture analysis to better understand the myocardial tissue differences between boys with DMD and healthy controls.

## Limitations

The study limitations include the general, well-known limitations related to myocardial T_1_ mapping [36, 37]. Importantly, significantly faster heart rates were detected in the DMD group compared to the healthy control group. Generally, heart rates are high in DMD and might be related to deconditioning along with changes in cardiac output [38]. As boys with DMD develop advanced cardiomyopathy, angiotensin-converting enzyme inhibitors and beta-blocker therapies are prescribed to lessen the severity of symptoms. This study did not correct for therapy effects on T_1_ mapping results. In order to mitigate the heart rate dependencies on T_1_ mapping, the sequence parameters used in this study were within recommended guidelines [39, 40].

The CMR data obtained for this study was within known institution-specific ranges and followed very controlled protocols within and between sites. The discrepancy in post-contrast myocardial and blood pool T_1_ measurements between Site-A and Site-B maybe described, in part, by the contrast injection method used at each site. At Site-A, contrast was administered via contrast media autoinjector, while hand injection was the method of choice at Site-B. Kinetic measurements of the contrast injection were not acquired, thus it is not currently possible to further assess the individual contrast dynamics and their overall impact on the group-wise comparisons. This particular sub-analysis is further limited by the group sample sizes.

Recruiting subjects with a rare, complex genetic disease whose cardiac involvement is understudied, is a difficult task—even more so to recruit a well-matched (i.e. age, height, weight) control group. Therefore, this study is limited by its sample size, which further limits subgroup analyses. Herein, the control group did not undergo post-contrast CMR as this would generally be contraindicated and impractical.

## Conclusions

3T CMR native T1 demonstrates the ability to characterize myocardial differences in boys with DMD and healthy, age- and sex-matched controls. Additionally, post-contrast T_1_ and ECV estimates in boys with DMD distinguish LGE+ from LGE− myocardium. ROC analysis revealed that in all classification tasks, T_1_ mapping biomarkers outperform LVEF, a conventional biomarker. Importantly, both native and post-contrast T_1_ and ECV estimates are promising diagnostic CMR biomarkers for assessing myocardial remodeling in Duchenne muscular dystrophy.

## Supplementary information

**Additional file 1: Figure S1.** Example case from a boy with DMD showing the regions of interest (ROI) manually drawn on a mid-ventricular short-axis (A) pre-contrast/native and (B) post-contrast T1 map and (C) an extracellular volume (ECV) map. (D) The corresponding late gadolinium enhancement (LGE) image with areas of enhancement on the lateral free wall (arrows).

**Additional file 2: Figure S2.** Pre-contrast T1 as a function of LVEDVi (A) and LVMi (B) and Post-contrast T_1_ as function of LVEF (C) and LVESVi (D) in healthy controls (gray circles), LGE− (red diamonds) and LGE+ (green squares) boys with DMD. The red solid lines indicate the linear regression fit. Significant correlations are outlined by the dashed-lined rectangles. Significant correlations were observed in the LGE+ group only for the following: 1) native T_1_ and LVEDVi; 2) native T1 and LVMi; and 3) post-contrast T_1_ and LVEF. In LGE− boys with DMD, a significant correlation was observed between post-contrast T_1_ and LVEF only. No T_1_ and functional metrics were correlated in healthy controls.

## Data Availability

The datasets used and/or analyzed during the current study are available from the corresponding author on reasonable request.

## Abbreviations

AHA: American Heart Association
BMI: Body mass index
BSA: Body surface area
bSSFP: Balanced steady state free precession
CMR: Cardiovascular magnetic resonance
CNR: Contrast-to-noise ratio
DMD: Duchenne muscular dystrophy
ECG: Electrocardiogram
ECV: Extracellular volume fraction
HLA: Horizontal long axis
LGE: Late gadolinium enhancement
LV: Left ventricle/left ventricular
LVEDV: Left ventricular end diastolic volume
LVEDVI: Left ventricular end diastolic volume indexed to BSA
LVEF: Left ventricular ejection fraction
LVESV: Left ventricular end systolic volume
LVESVI: Left ventricular end systolic volume indexed to BSA
LVM: Left ventricular mass
LVMI: Left ventricular mass indexed to BSA
MOCO: Motion correction
MOLLI: Modified Look-Locker inversion recovery
PSIR: Phase sensitive inversion recover
ROC: Receiver operating characteristic
ROI: Region of interest
Sax: Short axis
SASHA: Saturation recovery single shot acquisition
SNR: Signal-to-noise ratio
T_1_: Longitudinal relaxation time constant
TE: Echo time
TI: Inversion time
TR: Repetition time
VLA: Vertical long axis
