# Supplementary figures and images for: T1-Mapping and extracellular volume estimates in pediatric subjects with Duchenne muscular dystrophy and healthy controls at 3T
Source: J Cardiovasc Magn Reson. 2020 Dec 10;22:85. doi: 10.1186/s12968-020-00687-z (PMC7731511; doi:10.1186/s12968-020-00687-z)

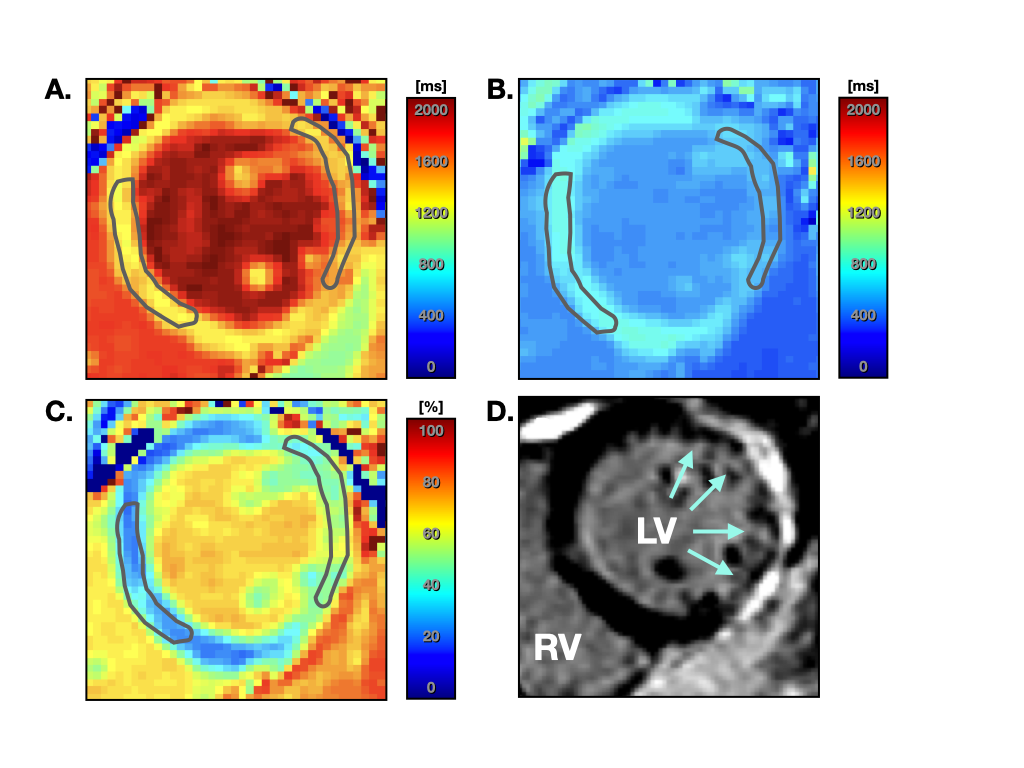

Supplement: Supplementary file 1 — Additional file 1: Figure S1. Example case from a boy with DMD showing the regions of interest (ROI) manually drawn on a mid-ventricular short-axis (A) pre-contrast/native and (B) post-contrast T1 map and (C) an extracellular volume (ECV) map. (D) The corresponding late gadolinium enhancement (LGE) image with areas of enhancement on the lateral free wall (arrows). [file 12968_2020_687_MOESM1_ESM.png]

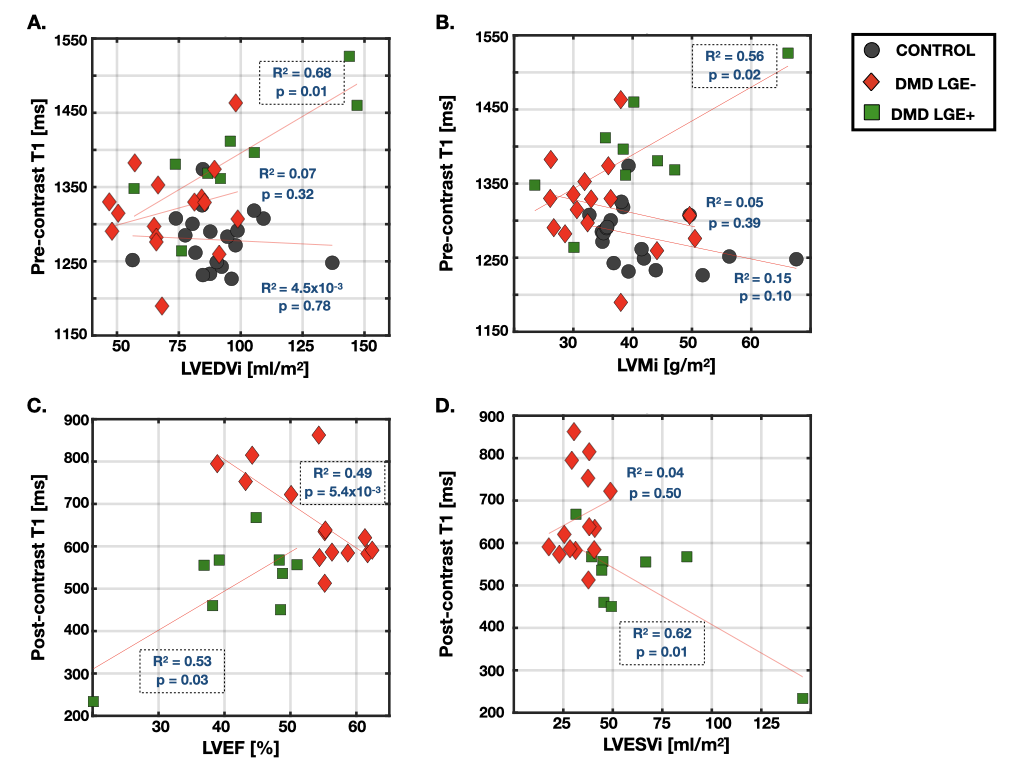

Supplement: Supplementary file 2 — Additional file 2: Figure S2. Pre-contrast T1 as a function of LVEDVi (A) and LVMi (B) and Post-contrast T1 as function of LVEF (C) and LVESVi (D) in healthy controls (gray circles), LGE− (red diamonds) and LGE+ (green squares) boys with DMD. The red solid lines indicate the linear regression fit. Significant correlations are outlined by the dashed-lined rectangles. Significant correlations were observed in the LGE+ group only for the following: 1) native T1 and LVEDVi; 2) native T1 and LVMi; and 3) post-contrast T1 and LVEF. In LGE− boys with DMD, a significant correlation was observed between post-contrast T1 and LVEF only. No T1 and functional metrics were correlated in healthy controls. [file 12968_2020_687_MOESM2_ESM.png]
